# A sodium-ion sulfide solid electrolyte with unprecedented conductivity at room temperature

**DOI:** 10.1038/s41467-019-13178-2

**Published:** 2019-11-20

**Authors:** A. Hayashi, N. Masuzawa, S. Yubuchi, F. Tsuji, C. Hotehama, A. Sakuda, M. Tatsumisago

**Affiliations:** Department of Applied Chemistry, Graduate School of Engineering, Osaka Prefecture University, 1-1 Gakuen-cho, Naka-ku, Sakai, Osaka 599-8531 Japan

**Keywords:** Electronic materials, Batteries

## Abstract

Solid electrolytes are key materials to enable solid-state rechargeable batteries, a promising technology that could address the safety and energy density issues. Here, we report a sulfide sodium-ion conductor, Na_2.88_Sb_0.88_W_0.12_S_4_, with conductivity superior to that of the benchmark electrolyte, Li_10_GeP_2_S_12_. Partial substitution of antimony in Na_3_SbS_4_ with tungsten introduces sodium vacancies and tetragonal to cubic phase transition, giving rise to the highest room-temperature conductivity of 32 mS cm^−1^ for a sintered body, Na_2.88_Sb_0.88_W_0.12_S_4_. Moreover, this sulfide possesses additional advantages including stability against humid atmosphere and densification at much lower sintering temperatures than those (>1000 °C) of typical oxide sodium-ion conductors. The discovery of the fast sodium-ion conductors boosts the ongoing research for solid-state rechargeable battery technology with high safety, cost-effectiveness, large energy and power densities.

## Abstract (web summary)

Solid-state rechargeable batteries using solid electrolytes instead of liquid ones could address the safety and energy density issues. Here the authors report a Na-ion solid electrolyte Na_2.88_Sb_0.88_W_0.12_S_4_ which exhibits record high ionic conductivity of 32 mS/cm at room temperature.

## Introduction

Development of all-solid-state rechargeable batteries has been desired because they have superior characteristics, such as a long cycle life, high safety, high-energy density, and high rate capability^1–4^. To realize bulk-type solid-state batteries using electrodes and electrolyte particles, superior solid electrolytes with high ionic conductivity and good ductility are desired, as they can facilitate wide contact areas with active materials simply upon pressing, without a high-temperature-sintering process^5^. Sulfide solid electrolytes meet this demand. Among solid electrolytes, Li_10_GeP_2_S_12_ (LGPS)-type crystalline sulfides^4,6^ show excellent Li-ion conductivity of over 10^−2^ S cm^−1^, which is higher than that of the conventional organic liquid electrolyte used in lithium-ion batteries, considering its low Li^+^ transference number (below 0.5). Since our discovery of cubic Na_3_PS_4_ with a relatively high Na ion conductivity of >10^−4^ S cm^−1^ in 2012^7^, studies for developing new Na-ion-conducting sulfides^8–22^ have rapidly increased. In 2016, a good Na-ion conductivity of 1 × 10^−3^ S cm^−1^ was achieved in Na_2.9375_PS_3.9375_Cl_0.0625_^10^ with Na vacancies and sodium antimony sulfide, Na_3_SbS_4_^11–13^. In addition, several other sulfides including Na_11_Sn_2_PS_12_^16,17^ and Na_3_SbSe_4_^18^ exhibit Na-ion conductivities higher than 10^−3^ S cm^−1^ at 25 °C. However, Na_10_GeP_2_S_12_ (NGPS)^22^ exhibits a moderate Na-ion conductivity of 10^−5^ S cm^−1^, which is much lower than the Li-ion conductivity of LGPS, implying that compositions that impart good Li-ion conductivity do not essentially impart good Na-ion conductivity.

Further, typical oxide crystalline electrolytes of NASICON^23^ and *β*-alumina^24^ sintered at high temperatures show a high Na-ion conductivity of 10^−3^ S cm^−1^. As for electrolytes with typical NASICON structure, the Na-ion conductivity in Na_3_Zr_2_Si_2_PO_12_^23^ is higher than the Li-ion conductivity in NASICON-type Li_1.3_Al_0.3_Ti_1.7_(PO_4_)_3_^25^, which implies that the Na-ion mobility in solid electrolytes is inherently higher than the Li ion mobility. This is because of the weaker Lewis acidity of Na ions, which have weaker electrostatic interaction with the oxide or sulfide anion species constituting the crystalline structure. However, Na-ion conducting sulfide electrolytes with the same degree of conductivity as that of the best Li ion conductor, LGPS, have not been discovered.

Here we report a sulfide Na-ion conductor with ionic conductivity higher than the best Li-ion conductivity of 2.5 × 10^−2^ S cm^−1^ in LGPS-type Li_9.54_Si_1.74_P_1.44_S_11.7_Cl_0.3_^4^. The sulfide superionic conductor with the composition of Na_2.88_Sb_0.88_W_0.12_S_4,_ obtained by replacing some of the antimony ions in Na_3_SbS_4_ by aliovalent tungsten ions, exhibits a room temperature conductivity of 3.2 × 10^−2^ S cm^−1^ in a sintered body; the conductivity is superior to those of any reported Li-ion and Na-ion conductors. In addition, the electrolyte shows good stability against hydrolysis, which is the origin of H_2_S gas evolution in an air atmosphere. The crystalline structure and electrical properties of Na_2.88_Sb_0.88_W_0.12_S_4_ are also demonstrated and discussed.

## Results

### Material synthesis and structure analysis

**Fig. 1:**
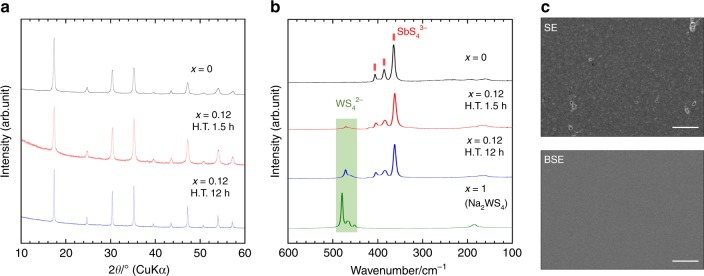
Structural data for Na_3−*x*_Sb_1−*x*_W_*x*_S_4_ (*x* = 0 and 0.12) electrolytes prepared by heat treatment (H.T.) at 275 °C for 1.5–12 h. **a** X-ray diffraction patterns, **b** Raman spectra, and **c** cross-sectional SEM images (scale bar: 1 μm) of the prepared Na_3−*x*_Sb_1−*x*_W_*x*_S_4_

A mechanochemical process using a planetary ball mill apparatus was used to directly produce crystalline Na_3_SbS_4_ (Supplementary Fig. 6a), and its crystallinity increased after heat treatment at 275 °C for durations ranging from 1.5 to 12 h. X-ray diffraction (XRD) patterns of the Na_3−*x*_Sb_1−*x*_W_*x*_S_4_ (*x* = 0 and 0.12) electrolytes prepared by heat treatment are shown in Fig. 1a. The three Raman bands around 360, 380, and 410 cm^−1^ in the Raman spectrum (Fig. 1b) of the prepared Na_2.88_Sb_0.88_W_0.12_S_4_ reveals the presence of SbS_4_^3−^ units, while the Raman band at ~470 cm^−1^ corresponds to the presence of WS_4_^2−^ units, which are typically observed for crystalline Na_2_WS_4_. A fairly weak peak at ~420 cm^−1^, probably due to WS_2_, is observed in the spectrum of Na_2.88_Sb_0.88_W_0.12_S_4_ heated for 1.5 h, while no peak is observed for that heated for 12 h. The Raman bands of the other starting materials (Sb_2_S_3_, Na_2_S, S_8_) are not observed in the spectra of Na_2.88_Sb_0.88_W_0.12_S_4_ (Supplementary Fig. 1). Cross-section of the pelletized Na_2.88_Sb_0.88_W_0.12_S_4_ heated at 275 °C for 12 h was observed by field-emission scanning electron microscopy (FE-SEM), and the secondary electron (SE) and backscattered electron (BSE) images are shown in Fig. 1c. As no composition-dependent contrast is observed in the BSE image, all the elements including tungsten are assumed to be uniformly dispersed in the monitored cross-section. In addition, energy-dispersive X-ray spectroscopic (EDX) mapping indicates the presence of tungsten in the entire SEM area (Supplementary Fig. 2). Elemental analysis using Rutherford backscattering spectrometry (RBS) predicts that the composition is Na_2.88_Sb_0.86_W_0.11_S_4_, and the error in the analysis of antimony is 0.86 ± 0.04. The experimentally determined composition is almost the same as the nominal composition of Na_2.88_Sb_0.88_W_0.12_S_4_.

**Fig. 2:**
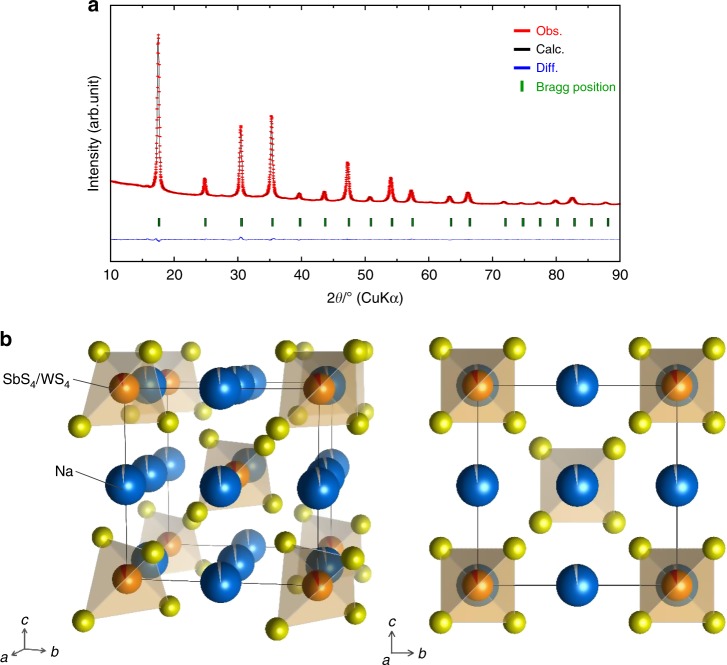
Crystal structure of Na_2.88_Sb_0.88_W_0.12_S_4_ heated at 275 °C for 12 h. **a** Rietveld refinement profiles of X-ray powder diffraction data for Na_2.88_Sb_0.88_W_0.12_S_4_. Red dots and black lines denote the observed and calculated XRD patterns, respectively. The green sticks mark the position of the reflections for Na_2.88_Sb_0.88_W_0.12_S_4_. The difference between the observed and calculated patterns is indicated by the blue line. **b** Crystal structure of cubic Na_2.88_Sb_0.88_W_0.12_S_4_ with the unit cell outlined. The Na, Sb, W, and S sites are represented by blue, orange, gray, and yellow balls, respectively. Na is linearly arranged, and distortion of the Sb_4_/WS_4_ tetrahedra is very small compared to the tetragonal structure of Na_3_SbS_4_ (Supplementary Fig. 3)

Rietveld refinement analysis was conducted for the powder XRD pattern of the prepared Na_2.88_Sb_0.88_W_0.12_S_4_ (Fig. 2a) and the results are shown in Supplementary Table 1. Na_2.88_Sb_0.88_W_0.12_S_4_ has a cubic structure (*a* = 7.1920(1) Å, *I-*43*m* (no. 217), and the unit cell consists of a body-centered sublattice with SbS_4_^3−^ and WS_4_^2−^ units, and tungsten occupies the antimony site (2*a*), as shown in Fig. 2b. The W occupancy is 0.078(5), which is smaller than the W content at the nominal composition. The EDX and RBS results show that Sb is replaced by W based on the nominal composition. The W occupancy mismatch is believed to be based on the quality of XRD data measured using a laboratory X-ray diffractometer. Three-dimensional (3D) conduction of Na ions is expected in this cubic structure. The occupancy of the Na site (6*b*) is 0.974(2), suggesting the creation of vacancies in the lattice. The relatively large atomic displacement factor (***B***) of Na may be related to the dynamic motion of Na ions, as observed in the cubic Na_3_PS_4_ phase^26^. Detailed structural analysis using synchrotron XRD is important to determine the W/Sb site occupancy and Na site vacancy in Na_2.88_Sb_0.88_W_0.12_S_4_. In contrast, Na_3_SbS_4_ (*x* = 0) has a tetragonal structure (*a* = 7.1708(12) Å, *c* = 7.2376(2) Å, *P-*42_1_*c* (no. 114)), which is in good agreement with the previous reports^11,12^; The value of *S* obtained by the Rietveld refinement of Na_3_SbS_4_ assuming a cubic phase was higher (i.e. *S = R*_wp_/*R*_e_ = 3.34) than that (*S* *=* 1.98) obtained assuming a tetragonal phase. Tetragonal Na_3_SbS_4_ has two Na sites and their occupancies are unity, as shown in Supplementary Fig. 3 and Supplementary Table 2. Na ion conduction along the *c-*axis in the tetragonal phase would be less likely than in the cubic phase^12^. It is noteworthy that the inclusion of tungsten into crystalline Na_3_SbS_4_ is effective in introducing Na vacancies in the lattice, and it facilitates the formation of a cubic phase, which can facilitate isotropic 3D fast-ion conduction.

### Conduction characteristics

**Fig. 3:**
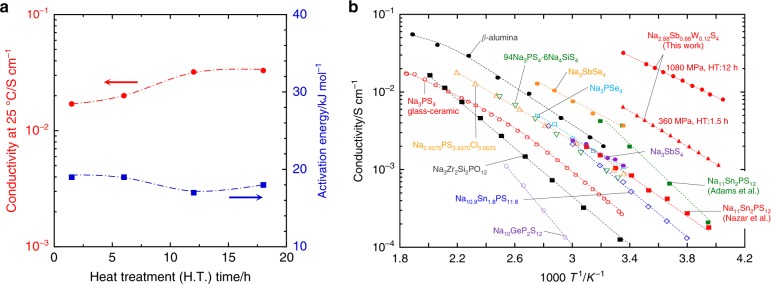
Conductive behavior of Na_2.88_Sb_0.88_W_0.12_S_4_ electrolyte. **a** Conductivity at 25 °C (*σ*_25_) and activation energy *E*_a_ for the conduction of the pelletized Na_2.88_Sb_0.88_W_0.12_S_4_ electrolytes as a function of heat treatment (H.T.) time at 275 °C. The pellets were prepared by cold-pressing the electrolyte powder at 1080 MPa, followed by heat treatment at 275 °C. **b** The Arrhenius plot of the Na ion conductivity of the Na_2.88_Sb_0.88_W_0.12_S_4_ electrolyte developed in this study and representative Na ion conductors reported so far

Conductivity of the pelletized Na_2.88_Sb_0.88_W_0.12_S_4_ was measured by the AC impedance method. Pellets of the material were prepared by cold-pressing the electrolyte powder at 1080 MPa, followed by heat treatment at 275 °C. Figure 3a shows its conductivity at 25 °C (*σ*_25_) and activation energy *E*_a_ for conduction as functions of the duration of heating at 275 °C. As an example, Nyquist plots at −25 °C for the pellets heated for 1.5 and 12 h are shown in Supplementary Fig. 4a, and the cross-sectional SEM image of the pellets are shown in Supplementary Fig. 4b. Only a spike owing to the capacitance at the interface between each electrolyte pellet and Au current collector is observed; other resistance components are not clearly detected in the high-frequency region even at a low measurement temperature of −25 °C. In this study, the total conductivity is thus determined from the resistance at the intersection of the spike and the *x*-axis; this resistance refers to the total resistance including both the grain-bulk and grain-boundary components of the electrolytes.

The pellet heated for 1.5 h (relative density of 93%) has *σ*_25_ of 1.7 × 10^−2^ S cm^−1^ and *E*_a_ of 19 kJ mol^−1^. The electronic conductivity at 25 °C measured by a DC polarization technique is 7.9 × 10^−8^ S cm^−1^ or lower, as shown in Supplementary Fig. 5, which is five orders of magnitude lower than the total conductivity mentioned above; the Na-ion transport number of the prepared Na_2.88_Sb_0.88_W_0.12_S_4_ is almost unity. Na-ion conductivity increased with an increase in the heating time as shown in Fig. 3a, because of the increase in the crystallinity of the cubic phase as well as the relative density of the pellet, as determined by the XRD analysis (Supplementary Fig. 6) and cross-sectional SEM imaging (Supplementary Fig. 4b). The best conducting behavior is obtained for a pellet with a relative density of 95% obtained after heating for 12 h, which shows the highest *σ*_25_ of 3.2 × 10^−2^ S cm^−1^ and the lowest *E*_a_ of 17 kJ mol^−1^.

Further, the conductivity of the material is definitely affected by the tungsten content (*x*) of Na_3−*x*_Sb_1−*x*_W_*x*_S_4_. Pellets of Na_3−*x*_Sb_1−*x*_W_*x*_S_4_ with varying *x* were prepared by cold-pressing the milled Na_3−*x*_Sb_1−*x*_W_*x*_S_4_ powders at 360 MPa, followed by heating at 275 °C for 1.5 h. XRD patterns of these pellets indicate the formation of cubic crystals without any impurity phases at all the compositions (Supplementary Fig. 7b). No apparent peak shift in the XRD patterns is observed with increasing *x*. This is probably because of the nearly same ionic radii of Sb^5+^ and W^6+^. The temperature-dependence of conductivities of the prepared pellets obeys the Arrhenius equation (Supplementary Fig. 6c). The conductivity increases and the activation energy decreases with an increase in *x*, and better conductive behavior is achieved for *x* ranging from 0.12 to 0.15 (Supplementary Table 3). The electrolyte with *x* = 0.12 gives the lowest *E*_a_ of 21 kJ mol^−1^ and a high conductivity of 6.4 × 10^−3^ S cm^−1^, which are superior to those of Na_3_SbS_4_ (*x* = 0) (*σ*_25_ of 2.1 × 10^−3^ S cm^−1^ and *E*_a_ of 26 kJ mol^−1^). Although this comparison is carried out among the lower density pellets prepared at a smaller molding pressure and a shorter heating time, the partial substitution of tungsten for antimony definitely improves the Na-ion conductivity of the Na_3_SbS_4_ electrolyte.

The Arrhenius plot of the Na-ion conductivity of the Na_2.88_Sb_0.88_W_0.12_S_4_ electrolyte developed in this study is shown in Fig. 3b; it shows the highest conductivity among Na-ion conductors reported so far. In addition, *σ*_25_ of 3.2 × 10^−2^ S cm^−1^ for the electrolyte is higher than that of the best LGPS-type Li_9.54_Si_1.74_P_1.44_S_11.7_Cl_0.3_ Li-ion conductor (*σ*_25_ = 2.5 × 10^−2^ S cm^−1^). Here, we have successfully discovered for the first time, a Na-ion-conducting sulfide electrolyte that has higher conductivity than all known Li-ion conductors.

### Exposure to the atmosphere

**Fig. 4:**
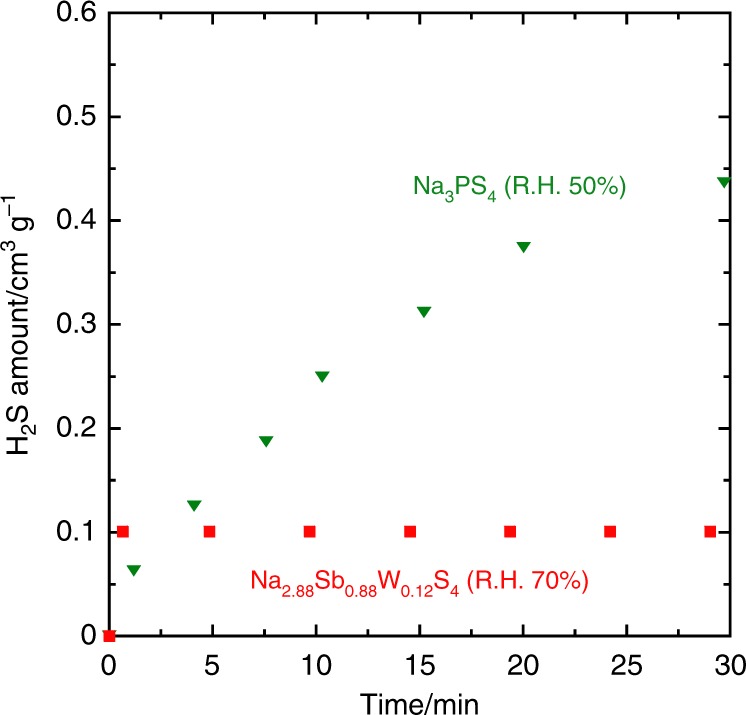
Monitoring H_2_S gas generated from Na_2.88_Sb_0.88_W_0.12_S_4_ as a function of exposure time to humid air (relative humidity (R.H.) = 70%). The H_2_S content is normalized by the weight of sulfur in the samples. For comparison, the same measurement was carried out for Na_3_PS_4_ under a less-humid condition of R.H. = 50%

Another advantage of the Na_2.88_Sb_0.88_W_0.12_S_4_ electrolyte is its high tolerance to air; that is, the amount of harmful H_2_S generated when it is exposed to air is almost negligible. H_2_S generation from the electrolyte even under a highly humid environment (relative humidity, R.H. of 70%) is suppressed when compared to that from a conventional Na_3_PS_4_ under a less-humid condition with R.H. of 50% (Fig. 4). An XRD pattern similar to that of the hydrate, Na_3_SbS_4_·9H_2_O is observed for the electrolyte after exposure to humid air (Supplementary Fig. 8). The formation of a hydrate compound without H_2_S generation has already been reported for crystalline Na_3_SbS_4_^11^; our tungsten-substituted sulfide conductor also undergoes a similar structural transformation with hydration.

## Discussion

In conclusion, a Na_2.88_Sb_0.88_W_0.12_S_4_ superionic conductor was developed. The conductivity of this material, 3.2 × 10^−2^ S cm^−1^ is the highest among sulfide Li and Na-ion conductors reported so far. The generation of Na vacancies and stabilization of the cubic phase by the partial substitution of antimony in Na_3_SbS_4_ with tungsten are responsible for its superior conduction. The Na_2.88_Sb_0.88_W_0.12_S_4_ electrolyte with high tolerance to H_2_S gas generation under an ambient atmosphere would improve safety of all-solid-state batteries and reduce manufacturing cost. The developed sulfide superionic conductor contributes to the realization of solid-state batteries with high-energy density and power density.

## Methods

### Material synthesis

Na_3−*x*_Sb_1−*x*_W_*x*_S_4_ solid electrolytes were prepared via a mechanochemical process using a planetary ball mill apparatus (Pulverisette 7, Fritsch GmbH). Reagent-grade Na_2_S (Nagao Co., 99.1%), Sb_2_S_3_ (Nihonseiko Co., 99.8%), WS_2_ (Aldrich Chem. Co., 99%), and S (Aldrich Chem. Co., 99.98%) powders were used as the starting materials. The starting materials were weighed at stoichiometric compositions of Na_3−*x*_Sb_1−*x*_W_*x*_S_4_ (e.g., the molar ratio of Na_2_S:Sb_2_S_3_:S = 50.0:16.7:33.3 for Na_3_SbS_4_ and the molar ratio of Na_2_S:Sb_2_S_3_:S:WS_2_ = 48.0:14.7:33.3:4 for Na_2.88_Sb_0.88_W_0.12_S_4_). A mixture of the starting materials (0.6 g) was ground using a mortar and a pestle for 10 min and placed in a 45 mL ZrO_2_ pot containing 250 ZrO_2_ balls of diameter 4 mm. Then, the starting materials were milled at a rotation speed of 510 rpm for 5 or 30 h. It was further subjected to heat treatment at 275 °C for several hours (1.5–18 h) in a porcelain crucible to obtain the final Na_3−*x*_Sb_1−*x*_W_*x*_S_4_ electrolytes. The colors of the prepared electrolytes Na_3_SbS_4_ and Na_2.88_Sb_0.88_W_0.12_S_4_ were brown and dark brown, respectively. All the steps were carried out in dry Ar atmosphere.

### Material characterization

The crystallographic phase was identified using an X-ray diffractometer (SmartLab, Rigaku) with Cu-*Kα* radiation. Diffraction data were collected in steps of 0.02° in the 2*θ* range 10−60° at a scan rate of 10° min^−1^. X-ray diffraction (XRD) measurements were performed using an airtight vessel with a beryllium window to prevent the sample from exposure to air. The crystalline structure was refined using the computer program PDXL2 (Rigaku Co.), and the crystal models were visualized using the VESTA software^27^. Rietveld refinement was performed for the XRD data collected in steps of 0.02° in the 2*θ* range 10−120° at a scan rate of 2° min^−1^. For this, first, the peak shape, background coefficient, scale factor, and lattice constants were refined. Then, the occupancy was fixed at the stoichiometric composition, and the atomic displacement factor was refined. Finally, the above parameters were fixed, and the occupancy, except for sulfur, was refined.

Raman spectroscopy was carried out on a Raman spectrophotometer (LabRAM HR-800, Horiba) equipped with a 532 nm He–Ne laser to identify the structural units. The electrolyte samples were placed in an airtight vessel filled with dry Ar gas, and Raman scattering signals from the samples were collected through a transparent quartz plate mounted on the upper side of the vessel.

Scanning electron microscopy was carried out on a FE-SEM (SU8220, Hitachi High-Technologies) equipped with an energy-dispersive X-ray spectroscopy system (EDX, EMAXEvolution X-MAX, Horiba Ltd.). The samples were transferred from an airtight vessel with dry Ar gas to the apparatus.

The chemical composition was determined by RBS. The RBS measurement was carried out with He^+^ beam accelerated to 2.3 MeV using customized RC-43 (National Electrostatics Co.). The sample was set in a high-vacuum chamber with the sample surface perpendicular to the ion beam. The beam current was measured with a Faraday cup and then tuned to 10 nA. Energy spectrum of the backscattered He^+^ was obtained using a surface barrier detector (SBD) set at 160°. The alpha rays emitted from the sample due to nuclear reactions were also detected with an SBD set at 146°. The RBS spectrum was collected for ~2 h under the measurement condition. The RBS revealed that the ball-milled sample contained about 0.06 wt% of Zr. The amount is, however, so less that the influence of Zr contamination from the ball-milling media on the properties of Na_2.88_Sb_0.88_W_0.12_S_4_ is almost negligible.

The amounts of H_2_S gas evolved from Na_2.88_Sb_0.88_W_0.12_S_4_ were measured using a H_2_S gas sensor (GBL-HS, JIKCO Co.) and a humidity controller (SCS01, I.E. Service Co.). The H_2_S gas evolved from Na_3_PS_4_ was also measured for comparison. A powder-compressed pellet (100 mg) was placed in a 2-L sealed container, where the air humidity and temperature were controlled to 70% and 24–26 °C, respectively. The amount of H_2_S was normalized by the mass of sulfur in the samples.

### Electrical characterization

The ionic conductivity of pelletized Na_2.88_Sb_0.88_W_0.12_S_4_ electrolytes was measured by the AC impedance method. The data were collected in the range of 10Hz–1M Hz using an impedance analyzer (1260, Solartron) with an applied AC voltage of 10–25 mV. The milled samples were cold pressed at 360 or 1080 MPa into pellets and then heated at 275 °C for several hours to obtain the final electrolyte pellets. The diameter and thickness of the pellets were 10 and ~1 mm, respectively. Gold current collectors were used to cover the entire surface of both sides of the pellet. The pellet was sealed in a laminate-type pouch cell to prevent air exposure. The conductivity was measured in the temperature range of ca. −25 to 25 °C, controlled with an ethanol-cooled bath and a constant temperature bath. As an example, three specimens of Na_2.88_Sb_0.88_W_0.12_S_4_ prepared by pressing at 360 MPa and heating at 275 °C for 1.5 h showed almost the same conductivities ranging from 6.4 × 10^−3^ to 6.7 × 10^−3^ S cm^−1^ at 25 °C (Supplementary Table 3). The activation energy for conduction was calculated from the slope of the Arrhenius equation, *σ* = *σ*_0_ exp(−*E*_a_/*RT*), where *σ* is the ionic conductivity, *T* is the absolute temperature, *σ*_0_ is the pre-exponential factor for ionic conduction, *E*_a_ is the activation energy for the ionic conduction, and *R* is the gas constant. The errors in activation energy can be considered to be small, because the *R*^2^ values (coefficient of determination) for the Arrhenius plots exceed 0.99.

The electronic conductivity was measured on electrolyte pellet samples via a DC polarization technique. The data were collected using a potentio/galvanostat (1287, Solartron) with the applied DC voltages ranging from 0.07 to 0.98 V at 25 °C.

## Supplementary information

Supplementary Information

## Data Availability

The data that support the findings of this study are available from the corresponding authors upon reasonable request.

## Supplementary information

**Supplementary information** is avaliable for this paper at 10.1038/s41467-019-13178-2.
