# Supplementary material for: A sodium-ion sulfide solid electrolyte with unprecedented conductivity at room temperature
Source: Nat Commun. 2019 Nov 20;10:5266. doi: 10.1038/s41467-019-13178-2 (PMC6868223; doi:10.1038/s41467-019-13178-2)
Supplement: Supplementary file 1 — Supplementary Information [file 41467_2019_13178_MOESM1_ESM.pdf]

Supplementary Information

A sodium-ion sulfide solid electrolyte with unprecedented conductivity at room temperature

A. Hayashi *et al.*

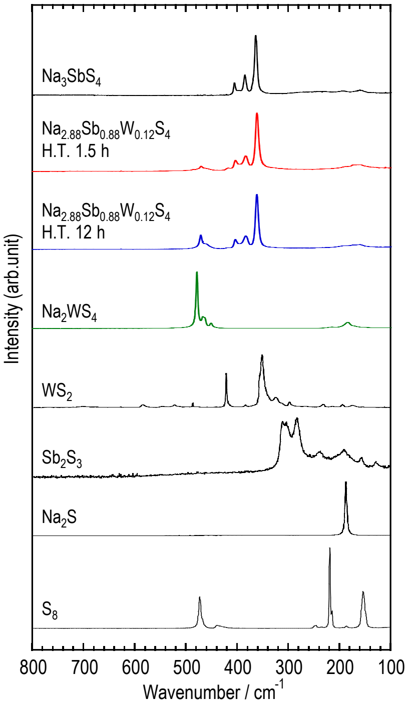

Supplementary Figure 1 Raman spectra of $Na_{2.88}Sb_{0.88}W_{0.12}S_4$ and its starting materials.

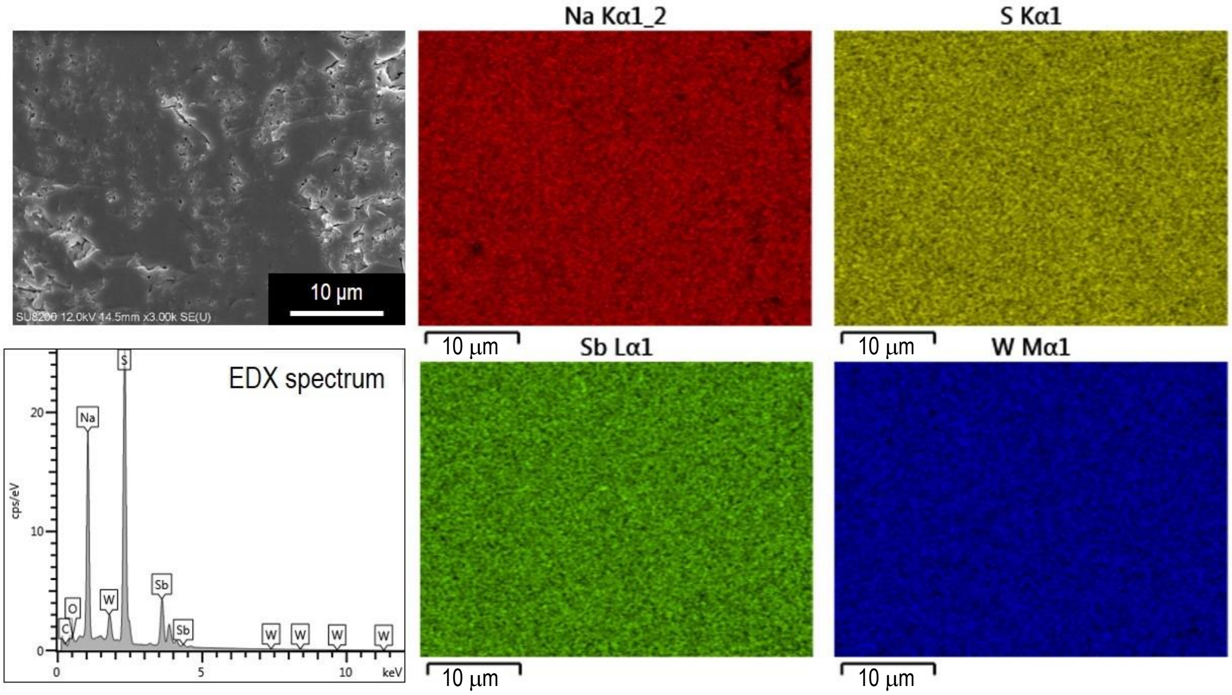

Supplementary Figure 2 SEM image and EDX mappings of Na, S, Sb, and W for the cross-section of a $Na_{2.88}Sb_{0.88}W_{0.12}S_4$ pellet.

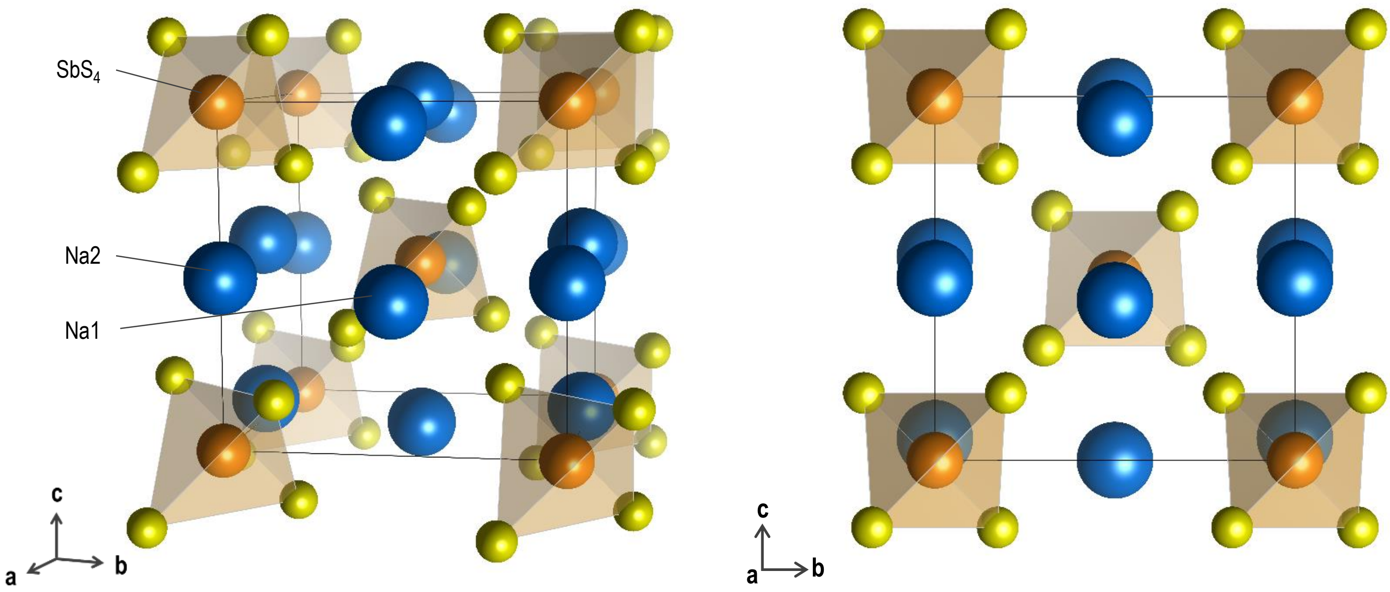

Supplementary Figure 3 Crystal structure of tetragonal Na_3SbS_4 (x = 0) with the unit cell outlined. The Na, Sb, and S sites are represented by blue, orange, and yellow balls, respectively. Na is arranged in a zigzag manner, and the Sb_4 tetrahedra is slightly distorted.

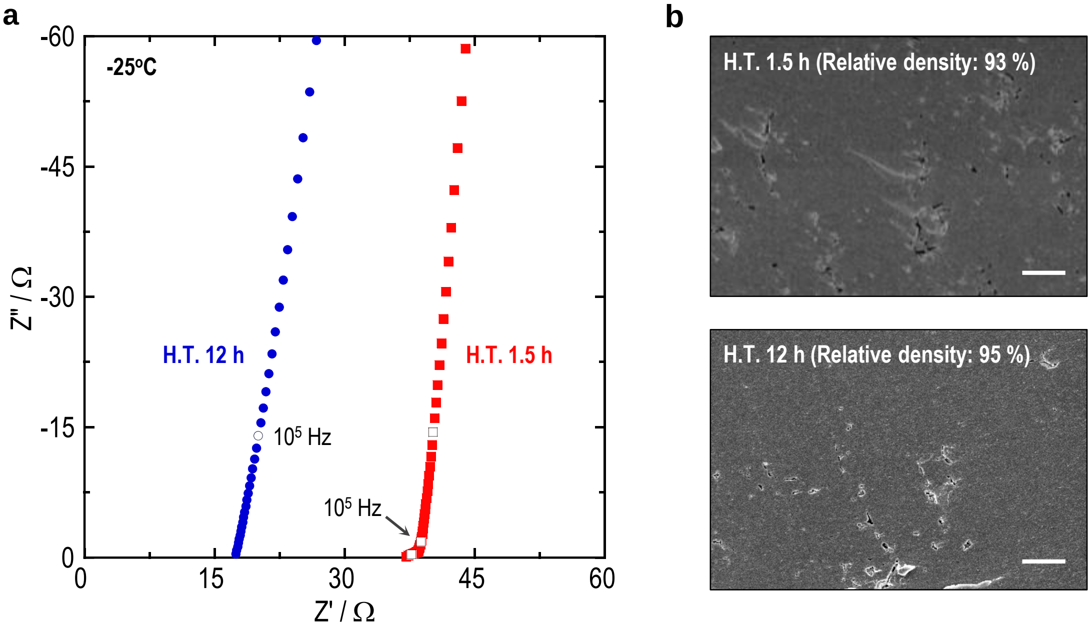

Supplementary Figure 4 Evaluation of electrical conductivity for $Na_{2.88}Sb_{0.88}W_{0.12}S_4$. (a) Nyquist plots at −25 °C for the $Na_{2.88}Sb_{0.88}W_{0.12}S_4$ pellets heated for 1.5 and 12 h. (b) Cross-sectional SEM images of the pellets. Scale bar is 2 μm for the top panel and 1 μm for the bottom panel.

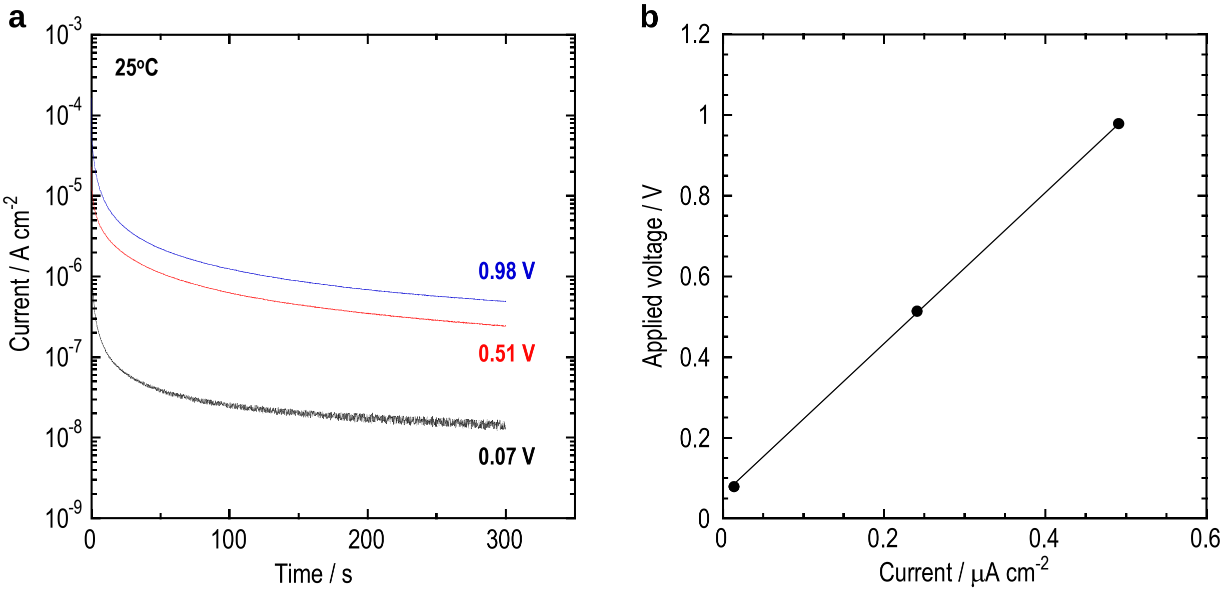

Supplementary Figure 5 Evaluation of electronic conductivity for $Na_{2.88}Sb_{0.88}W_{0.12}S_4$. (a) DC polarization curves for $Na_{2.88}Sb_{0.88}W_{0.12}S_4$ under the applied voltage of 0.07–0.98 V at 25 °C. (b) The relationship between the applied voltage and constant current is also shown.

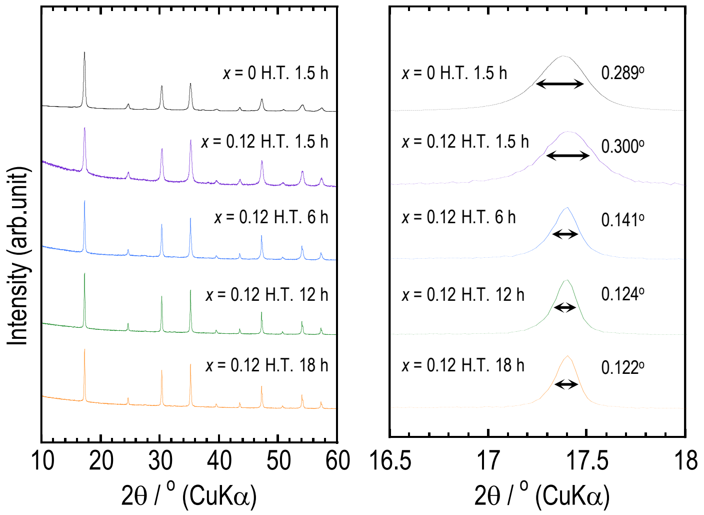

Supplementary Figure 6 XRD patterns of $Na_{3-x}Sb_{1-x}W_xS_4$ electrolytes prepared by heat treatment (H.T.) for different durations. Full-width at half maximum (FWHM) of the most intense peak is denoted in the right panel.

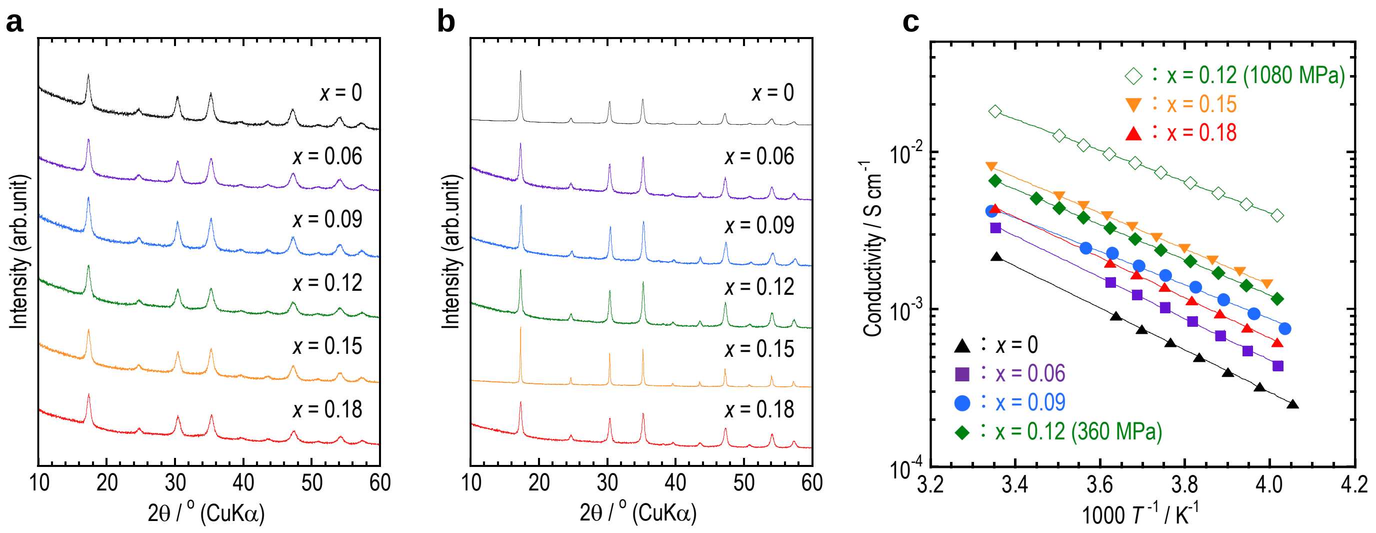

Supplementary Figure 7 XRD pattern and conductivity for the $Na_{3-x}Sb_{1-x}W_xS_4$ electrolytes prepared under different experimental conditions. (a) XRD patterns of milled $Na_{3-x}Sb_{1-x}W_xS_4$ samples and (b) those of the heat-treated $Na_{3-x}Sb_{1-x}W_xS_4$ samples. Heat treatment was carried out at 275 °C for 1.5 h for the milled samples pelletized with a molding pressure of 360 MPa. Temperature-dependence of the conductivity of the heat-treated pellets (b) is shown in panel (c). The Arrhenius plot for the pellet prepared with a molding pressure of 1080 MPa, followed by heating at 275 °C for 1.5 h is also displayed.

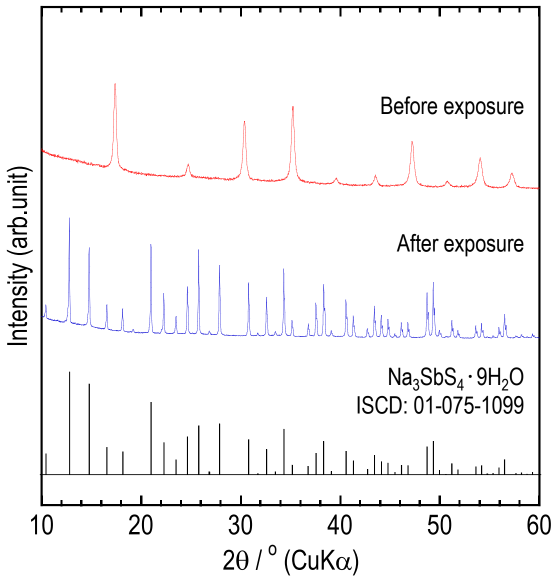

Supplementary Figure 8 XRD patterns of $Na_{2.88}Sb_{0.88}W_{0.12}S_4$ electrolytes before and after exposure to humid air for 1.5 h.

Supplementary Table 1 Crystallographic data for the prepared $Na_{2.88}Sb_{0.88}W_{0.12}S_4$.

**Crystal System**	Cubic	**Lattice Parameter**	a = 7.1921(1) Å
**Space Group**	$I\bar{4}3m$ (No. 217)	**Volume, *Z***	V = 372.02 Å^3, Z = 2

Atoms	Wyckoff	Occ.	*x*	*y*	*z*	B / Å^2
Na	6*b*	0.974 (2)	0	1/2	1/2	6.682
Sb	2*a*	0.922 (5)	0	0	0	0.984
W	2*a*	0.078 (5)	0	0	0	= ***B***(Sb)
S	8*c*	1	0.1851(1)	0.1851(1)	0.1851(1)	1.909

*R_{wp} = 0.0240, R_p = 0.0182, R_e = 0.0140, $S = R_{wp}/R_e$ = 1.71

Supplementary Table 2 Crystallographic data for the prepared Na_3SbS_4.

**Crystal System**	Tetragonal	**Lattice Parameter**	a = 7.1708(12) Å<br>c = 7.2376(2) Å
**Space Group**	$P\,\bar{4}2_1c$ (No.114)	**Volume, *Z***	V = 372.16 Å^3, Z = 2

**Atoms**	**Wyckoff**	**Occ.**	***x***	***y***	***z***	***B* / Å^2**
Na 1	4*d*	1	0	1/2	0.4396(5)	4.50(12)
Na 2	2*b*	1	0	0	1/2	4.6(2)
Sb	2*a*	1	0	0	0	1.13(2)
S	8*e*	1	0.3029(3)	0.3255(3)	0.6848(2)	1.82(4)

*R_{wp} = 0.0245, R_p = 0.0177, R_e = 0.0124, $S = R_{wp}/R_e$ = 1.98

Supplementary Table 3 Conductivity at 25 °C (σ_{25}) and activation energy (E_a) for conduction of the $Na_{3-x}Sb_{1-x}W_xS_4$ pellets prepared by cold-pressing at 360 MPa, followed by heat treatment at 275 °C for 1.5 hours.

$Na_{3-x}Sb_{1-x}W_xS_4$	σ_{25} / S cm^{-1}	E_a / kJ mol^{-1}
$x = 0$	2.1×10^{-3}	26
$x = 0.06$	3.4×10^{-3}	25
$x = 0.09$	4.4×10^{-3}	21
$x = 0.12$	6.4×10^{-3}	21
$x = 0.15$	7.7×10^{-3}	22
$x = 0.18$	4.5×10^{-3}	24
